# Understanding the Determinants Influencing Self-Medication with Antibiotics Among Malaysian Residents: A Qualitative Study to Inform Preventive Public Health Strategies

**DOI:** 10.3390/antibiotics13111070

**Published:** 2024-11-11

**Authors:** Adeel Aslam, Shazia Jamshed, Che Suraya Zin, Norny Syafinaz Ab Rahman, Syed Imran Ahmed, Zita Lívia Szabó, Márió Gajdács

**Affiliations:** 1 Faculty of Pharmacy and Biomedical Sciences, Mahsa University, Jenjarom 42610, Malaysia; 2 Department of Pharmacy Practice, School of Pharmacy, International Medical University, Kuala Lumpur 57000, Malaysia; 3 Department of Pharmacy Practice, Kulliyyah of Pharmacy, International Islamic University Malaysia, Kuantan 25200, Malaysia; 4 School of Pharmacy, College of Science, University of Lincoln, Lincoln LN6 7TS, UK; 5 Department of Oral Biology and Experimental Dental Research, Faculty of Dentistry, University of Szeged, 6720 Szeged, Hungary

**Keywords:** self-medication, antibiotics, non-prescription, antibiotic consumption, antimicrobial resistance, qualitative study

## Abstract

Introduction: Self-medication with antibiotics (SMA)—the use of these drugs without a physician’s diagnosis, supervision, or a medical prescription—has emerged as a significant crisis in many societies, being a particular concern in low- and middle-income countries. SMA practices hinder global efforts to reduce antibiotic consumption in the human health sector and to control antimicrobial resistance (AMR). The aims of the study were to explore the motivations, perspectives, and personal experiences of the Malaysian population related to SMA, analyzing their subjective viewpoints, attitudes, and behaviors. Methods: The present qualitative study applied an interpretivism paradigm to explore the complex topic of SMA; the interview questions were developed and confirmed by experts in AMR and pharmacy practice. Qualitative data analysis was carried out through a thematic approach. Results: Out of twenty-seven (*n* = 27) eligible individuals, eleven (*n* = 11) agreed to participate in in-depth interviews. Three main themes and seven subthemes were identified. Participants revealed directly purchasing antibiotics from pharmacies, due to doctors prescribing similar medications for comparable illnesses in the past. Trust issues related to physicians prescribing unnecessary additional medications and suspected personal motives were revealed. Budgetary constraints, timesaving, and aspects of self-care were identified as some of the key drivers for SMA. Conclusions: The current study’s findings contribute to the understanding of the complexities surrounding SMA and provides insights into the public perceptions and behaviors regarding unregulated antibiotic use in Malaysia. Understanding these dynamics may inform targeted public health interventions to address SMA to mitigate the development of AMR.

## 1. Introduction

Antibiotics play a pivotal role in the treatment and, in certain contexts, the prevention of bacterial infectious diseases, and they are some of the most effective tools in reducing morbidity and mortality associated with infectious pathologies [1,2,3]. However, the misuse, overuse, and inappropriate utilization of antibiotics have led to the emergence of antibiotic resistance (ABR), a subset of the global challenge of antimicrobial resistance (AMR), posing a significant global public health concern [4]. AMR is directly responsible for an estimated 1.27 million deaths annually, while approximately 4.95 million deaths are associated with bacterial AMR globally [5]. This burden is particularly severe in low- and middle-income countries (LMICs), where healthcare systems struggle to manage resistant infections effectively [6].

The prevalence of AMR has reached alarming levels worldwide, primarily driven by factors, such as incorrect use of antibiotics, including self-medication, dosage errors, and excessive consumption outside their indicated use [7,8]. Self-medication is characterized by the procurement and use of medicines by bypassing primary healthcare services and without consulting a physician, usually to manage acute symptoms of self-diagnosed illnesses [9]. Self-medication with antibiotics (SMA), encompassing the use of antibiotics without professional diagnosis, opinion, prescription, or supervision of a trained healthcare professional, has emerged as a significant crisis in many societies [6,10]. SMA is particularly prevalent in LMICs, where the public has limited access to healthcare (or where a government scheme does not subsidize healthcare), and where the availability of non-prescription antibiotics has made SMA a viable and common alternative due to its affordability [11,12]. On one hand, self-medication is often seen by its users as a part of self-care. However, in the case of SMA, it poses risks to patients’ health on an individual level (unnecessary exposure to antimicrobials may lead to severe adverse events), while on a global level, it contributes to the emergence of ABR and the spread of drug-resistant bacteria [13,14]. In Malaysia, as in many other Southeast Asian countries, the rising trend of multidrug-resistant (MDR) bacteria, such as *Escherichia coli*, *Klebsiella pneumoniae*, and *Staphylococcus aureus* poses, significant public health concerns. Local epidemiological data have shown increasing resistance patterns, particularly among Gram-negative pathogens, contributing to rising mortality and morbidity rates [15].

SMA practices hinder global efforts to reduce antibiotic consumption in the human health sector, streamline administration, and control ABR [16]. The sale of antibiotics without a prescription has proliferated, particularly in LMICs with weak or non-existent regulation and enforcement [17]. Furthermore, in these countries, the surveillance of ABR is also insufficient, further exacerbating the concerns of AMR on a state level [18]. Studies from Poland [19], Kosovo [20], Ghana [21], Nepal [21], and Pakistan [22] have consistently shown that antibiotics are frequently used without prescriptions for inappropriate purposes, particularly to treat symptoms related to upper respiratory tract infections, such as the common cold and other viral conditions, where antibiotics are ineffective. The World Health Organization (WHO) has emphasized the importance of antimicrobial stewardship, public awareness campaigns, and surveillance systems in its Global Action Plan on AMR as critical components of the efforts to reduce AMR worldwide [23].

The Malaysian Ministry of Health (MMH) reported a 16% annual increase in the usage of prescribed and over-the-counter antibiotics overall, as documented in the Malaysian Statistics on Medicine 2009–2010, highlighting the concerns related to their injudicious use [24,25]. A survey conducted in Alor Setar, Kedah, Malaysia, has revealed that 50% of respondents engaged in SMA [26]. Previously, a quantitative, questionnaire-based study was conducted to assess the prevalence and causes of SMA among Malaysian community-dwelling adults, finding a relatively low prevalence of SMA (15.1%) [27]. However, while these quantitative studies provided valuable prevalence data, they were unable to explore the underlying causes and motivations behind the practice of SMA among Malaysian adults, leaving a gap in understanding the behavioral and contextual factors contributing to this practice. Given the severity of SMA in the Malaysian context, it is crucial to improve our understanding and to gather insights into the choices and behaviors regarding the selection, acquisition, and use of over-the-counter antibiotics for self-medication, particularly the underlying socio-demographic and economic aspects; public health and health policy experts may use such data to design effective behavioral interventions to enhance the awareness of the public related to the threat of AMR. Reducing SMA and improving community-based antibiotic stewardship are essential steps in curbing the spread of MDR bacteria, which are becoming increasingly prevalent in Malaysia, as well as globally [28].

Thus, to comprehensively address the severity of SMA in the Malaysian context and to understand the factors influencing this behavior, particularly socio-demographic and economic aspects, the present study aimed to provide insights into the current prevalence and trends of SMA among Malaysians, as well as attitudes and knowledge related to antibiotics and SMA with a qualitative approach, using in-depth interviews. The study aims to explore the motivations, perspectives, and personal experiences of individuals related to SMA, analyzing their subjective viewpoints, attitudes, and behaviors. By addressing gaps in the existing literature, such as low awareness regarding the dangers of and inadequate behavior associated with SMA, this research aims to overcome the limitations of previous studies conducted in Malaysian communities.

## 2. Results

### 2.1. Socio-Demographic Characteristics

Twenty-seven (*n* = 27) individuals meeting the interview criteria were selected, but only eleven (*n* = 11) agreed to participate in the qualitative interview phase of the study. The most common reason for refusal was being too busy, and some participants did not reply to our request. The socio-demographic characteristics of our participants (*n* = 11) are summarized in Table 1. The majority (7/11) were male, with a median age of 36 years (range: 24–44). All participants in this study were permanent Malaysian residents. Most individuals were born in Malaysia and were of Malay ethnicity (*n* = 5), while the rest were Chinese (*n =* 2), Tamil (*n =* 2), and Arab (*n =* 2). Regarding their family situation, most (11/12) were married and had at least one child (9/12). Overall, 4/11 had an undergraduate education, 3/11 earned below 3000 Malaysian Ringgit (MYR) per month (for reference ~<652 USD), 5/11 earned between 3000–5000 MYR (for reference ~652–1087 USD), while 3/11 earned over 5000 MYR (for reference ~>1087 USD); for comparison, the mean monthly salary/wage at the time was 2804 MYR~610 USD, according to the Malaysian government.

### 2.2. Principal Findings, Themes, and Codes

Findings from the semi-structured interviews are presented in the subsequent subsections, along with themes, subthemes, and coding, as shown in Table 2. Texts or passages were marked if they appeared to align with the established categories or codes upon the initial reading of the transcripts by researchers. These highlighted texts were then coded using the existing categories. If any responses could not be assigned to the existing categories or codes, they were allocated a new category or code. Participant quotes were also included, when necessary, to provide context for the responses; when presenting quotes, additional information related to the context of specific words in the quotes was provided, which is enclosed in square brackets.

### 2.3. Theme 1: Practices Towards SMA

Participants in the current study had diverse perspectives on SMA; nevertheless, nearly all participants who reported partaking in SMA in the initial, quantitative phase of the study expressed a positive attitude towards this practice, as they believed that antibiotics are effective in treating their minor illnesses.

*“I usually use antibiotics to treat simple symptoms like sore throat, and sometimes, I also use them to alleviate fever, and even sometimes for body pains.”*

(I-1)

Among the participants who engaged in SMA, two main reasons were identified: Firstly, they believed that treating minor illnesses with antibiotics could prevent further health issues and the exacerbation of their condition. Secondly, some participants expressed that SMA could also reduce the risk of overprescription of these drugs. They believed that doctors often prescribe unnecessary additional medications (e.g., dietary supplements, decongestants, anti-inflammatory drugs) alongside antibiotics, and by engaging in SMA, they may avoid this perceived overprescription of other medicines.

*“I practice self-medication because I often don’t trust my doctors, as they sometimes prescribe unnecessary medications and supplements, such as multivitamins that are not needed.”*

(I-11)

The financial aspect emerged as the second major reason associated with non-prescription antibiotic usage. The majority of respondents revealed that they resorted to SMA for personal reasons, primarily due to financial constraints arising from limited funds for out-of-pocket healthcare expenditures or a lack of health insurance coverage.

*“My top priority is to support my family, and my earnings are not sufficient. Therefore, the high cost of medical treatment is a significant factor that motivates me to engage in SMA.”*

(I-3)

Moreover, the concern over the cost of antibiotics and medical consultations became particularly pronounced among participants who had a lower income, worked daily wage jobs, or were engaged in manual labor, a perspective which was echoed by several interviewees.

*“Yes…, it’s money-saving, and it is a matter of fact.”*

(I-5)

*“Yes…, cost is the biggest concern for me… I try to save as much money as I can… I also didn’t have health insurance, so for me, money is the biggest reason for SMA.”*

(I-3)

Furthermore, a lack of time emerged as a significant factor contributing to SMA. Participants, particularly those who are daily wage earners, noted that long waiting times for medical consultations and the challenges associated with travelling for healthcare often lead them to opt towards non-prescription antibiotic use. For these individuals, saving time directly supports their ability to work and earn a living, making SMA a practical, time-efficient alternative.

*“If I have to travel for my check-up, maybe I get stuck in traffic. So…, I do SMA to save time, and that’s most important to me…,”*

(I-3)

*“I participate in SMA because it is time saving; if I went for a check-up at my general practitioner’s office, it would take a lot of time”*

(I-7)

Additionally, it is worth noting that most participants were able to recall the (brand) names of antibiotics they had used previously. Curiously, the names of some specific medications are remembered more clearly by patients, while other names might slip away.

*“I remember using a couple of antibiotics when I fell ill in the past—XXX (a brand name for ciprofloxacin) and YYY (a brand name for metronidazole); those are the two names that stand out to me.”*

(I-3)

*“I used XXX (a brand name for combination of amoxicillin and clavulanic acid), YYY (a brand name for combination of amoxicillin and clavulanic acid), and ZZZ (a brand name for metronidazole) before to treat infections when I was sick.”*

(I-10)

In contrast, some participants of the current study faced difficulty in remembering the (brand) names of antibiotics they had used previously. Thus, they resorted to using a different technique: remembering the color and wrapper symbols of antibiotics.

*“I couldn’t remember the names of antibiotics, because their names are difficult to memorize. But I remember some antibiotic [boxes] due to their colors and sometimes with wrapper symbols.”*

(I-11)

Participants provided an interesting insight into the numerous ways that people manage to obtain antibiotics without a prescription from pharmacies and vendors, such as chemists’ shops. It appears that familiarity with a staff member inside the pharmacy often smooths the path to making such purchases, suggesting that relationship building with pharmacy staff plays a significant role in encouraging SMA. Additionally, some participants relied on their friends employed in various healthcare institutions, such as hospitals, to provide them with antibiotics. Moreover, a few of the participants mentioned that they sometimes reuse their previous prescriptions to acquire antibiotics once again.

*“To buy antibiotics, all it takes is a friendly chat with the right person… I even have two friends working at the pharmacy, and they’re always my first point of call”.*

(I-4)

*“I don’t have a specific place where I buy antibiotics. A friend who works in a hospital often supplies me with medicines [antibiotics]… Whenever I’m unwell, I call him up, and he brings me what I need.”*

(I-1)

*“Most of the time, I use the prescriptions I received from my doctor a long time ago to get more of the same antibiotics from the pharmacy.”*

(I-11)

In certain instances, participants acquired antibiotics from their home countries and brought them along with them when they travelled. Alternatively, they may ask friends or family members visiting their current residence to bring the medications for them. This is because buying antibiotics without a medical examination may be comparatively easier in their home countries. As explained by one participant:

*“I usually bring antibiotics from my home country, and sometimes I request friends or family members travelling here to bring me the medicine… Buying antibiotics without a medical check-up is effortless over there.”*

(I-8)

Interestingly, a unanimous assertion from all interviewees indicated a positive disposition towards SMA as a cost-saving strategy. The evidence suggests a prevalent trend among participants to procure antibiotics from pharmacies, drug stores, chemist shops, or even friends as an effort to reduce healthcare expenses. Moreover, interviewee reports also revealed that business owners and self-employed individuals tended to view SMA as a time-saving advantage rather than a cost-saving measure. It emerged that these participants considered the time dedicated to attending medical check-ups as a potential threat to their business engagements. Nevertheless, for some participants, convenience was the primary motivator for adopting SMA. From these admissions, it is evident that, for some, time and accessibility held more sway than purely monetary concerns when making the decision to partake in SMA.

*“Yes…, for sure, SMA saves my money and most importantly time. If I have to travel, I might get stuck in traffic, and my shop remains closed for that whole time, and I could lose a lot of money.”*

(I-1)

Another participant stated that:

*“Yes, you may say SMA saves money, but honestly speaking, I didn’t care about money. I use antibiotics because I find it convenient.”*

(I-6)

### 2.4. Theme 2: Attitudes Towards SMA

The participants’ responses reflect a concerning uncertainty regarding their previous experiences with antibiotics, which significantly influences their attitudes towards SMA. Many expressed confusion about whether they had completed their prescribed dosage regimens, with one participant stating:

*“I don’t remember whether I completed my dosage regimen in the past or not.”*

(I-8)

Another participant agreed, saying:

*“I am not sure about the dosage I should have followed previously, or whether I completed the antibiotic course before or not.”*

(I-10)

This lack of clarity suggests that participants may not fully understand the importance of adhering to prescribed antibiotic courses. This could lead to the misuse of leftover medications and contribute to the practice of SMA. Their attitudes towards SMA seem to be influenced by a combination of uncertainty and past experiences, highlighting the need for improved education on the appropriate use of antibiotics and the significance of completing prescribed treatments. Additionally, when participants were asked how often they had engaged in SMA in the past, most could not recall the exact number. However, those who experienced seasonal allergies or symptoms, such as cold, flu, and fever, reported using antibiotics approximately two to three times in the previous year.

*“I often suffer from seasonal allergies, especially during the rainy season, which usually leads to flu and a sore throat. So, I may have used antibiotics twice in the past three months.”*

(I-9)

*“I don’t recall the exact number of times I’ve used antibiotics for myself, but whenever I have a cold, flu, or fever, I typically resort to antibiotics. I estimate it to be around 3 to 4 times a year.”*

(I-3)

### 2.5. Theme 3: Knowledge Related to SMA

One of the concerns regarding the inappropriate use of antibiotics was the potential consequences that can arise from not completing the full course, taking the wrong dosage, or overconsumption. These reasons are often associated with beliefs about the relationship between ABR and SMA practices. Many participants in the study reported that they only use antibiotics for a period of two to three days or three to five days, and once their symptoms improve, they discontinue the use of the drug.

*“When I start taking antibiotics, after two to three days… I start feeling okay… then I stop using the antibiotics.”*

(I-7)

*“As soon as I start taking antibiotics, my symptoms improve. Usually, it takes three to five days to feel fine, and after that, I stop taking antibiotics.”*

(I-9)

Participants reported that they typically use antibiotics to treat minor ailments, such as colds, the flu, and sore throats. They stated that their course of antibiotic treatment usually lasts until they begin to feel better, at which point they discontinue the medication. Additionally, some participants also mentioned that they used antibiotics as a preventive measure against further illness or to prevent worse symptoms from occurring.

*“I generally resort to antibiotics for ailments, like cold, flu, and sore throat. I continue the course until my health improves. I believe antibiotics help in preventing any further illness.”*

(I-9)

Additionally, a number of participants mentioned that the duration of their recovery was not fixed and significantly depended on the severity of their condition. Participants claimed that if the ailment was minor, their recovery was relatively quick; whereas, more severe conditions required more time for the symptoms to improve. One participant interestingly mentioned that the quality of antibiotics also plays a significant role in recovery duration. Out of all the study participants, only two reported experiencing adverse effects associated with SMA.

*“There isn’t a set recovery time period. My illness may improve within a few days, such as two to three days, or it might take a week.”*

(I-2)

*“The time it takes for me to recover really depends on the severity of my illness. Sometimes it’s as quick as two to three days, and other times it may take longer.”*

(I-5)

*“Sometimes, after taking antibiotics, I notice a bitter taste in my mouth and a change in taste perception.”*

(I-3)

*“Usually, when I start taking antibiotics, I experience a change in taste in my mouth, and it’s an unpleasant experience.”*

(I-8)

Interestingly, most participants in the current study, all of whom were practicing SMA, demonstrated an understanding of antibiotics or AMR. Such knowledge potentially increases the likelihood of the appropriate use of antibiotics as well. The study participants gained their knowledge of these issues from various sources, including family members, friends, and the Internet.

A 30-year-old male participant from Malaysia was quite sure about his understanding of ABR. He described it as a condition where a drug takes longer than usual to produce its intended effect when used repeatedly over time:

*“Yes…, for sure I know about ABR; it may be defined as a condition where a drug, when used repeatedly over a period, takes more time than usual to exert its effect.”*

(I-5)

This participant highlighted their awareness about ABR, alongside their knowledge of drug resistance. The participant has also added that their comprehension of these topics is fortified by their habit of searching and reading online resources related to public health:

*“Yes, my brother is well-informed about topics, such as ABR and drug resistance. As I mentioned earlier, I actively search and read online websites that cover public health matters.”*

(I-5)

Moreover, a 37-year-old participant mentioned his wife, a pharmacist, as his primary source of AMR-related knowledge. He said she warned him that certain medications might cause diarrhea and also informed him about the concept of ABR:

*“My wife informed me that certain medications have the potential to cause diarrhea. Additionally, she educated me about the concept of resistance.”*

(I-6)

Lastly, a 40-year-old participant acknowledged his family’s medical background as a major influence on his understanding of ABR. He remembered being told that repetitive use of antibiotics could “lead to the body becoming resistant to them”:

*“My family has a medical background, so they often provide me with various pieces of information. Although I cannot recall everything, one concept that stands out is ABR, which occurs when our bodies develop resistance after prolonged use of antibiotics.”*

(I-11)

In general, all of the study participants agreed that SMA is a positive approach and appropriate for self-care. The participants cited various motivations for this perspective. Four primary reasons emerged as significant motivators for those participants who considered SMA as a progressive move towards self-care. Firstly, they mention that they use SMA to treat minor illnesses, which prevents them from having worse symptoms.

*“SMA… is good, I… save money, and most prominently, it prevents me from having a more severe disease.”*

(I-1)

*“Yes…, SMA is a good step for self-care. When illness begins, and I start using antibiotics at once…, it saves me from worse symptoms.”*

(I-5)

Secondly, they illustrated the use of antibiotics for self-treatment of minor ailments as a preventive measure against developing severe disease.

*“SMA is beneficial. It helps me save money, and most importantly, it prevents me from contracting severe diseases.”*

(I-4)

*“Yes, SMA is indeed a good step towards self-care. When early symptoms of an illness appear, and I start using antibiotics promptly, it shields me from developing severe illnesses.”*

(I-7)

Thirdly, participants indicated saving costs, avoiding time wasted on long waiting periods for medical check-ups, travelling, and concerns related to language barriers as factors that persuade them to believe SMA is a beneficial approach towards self-care. Lastly, the experience of participants in using antibiotics, both prescribed and non-prescribed, has fostered practical knowledge. This knowledge spans understanding specific diseases or symptoms treatable with antibiotics, recognizing various antibiotics, and comprehending the duration necessary for antibiotic use. These experiences further bolster their confidence in practicing SMA.

*“I use antibiotics on my own to treat my disease, and I feel confident about this practice.”*

(I-3)

*“SMA is good if the individual is aware of what they are consuming and the implications of their actions.”*

(I-6)

*“SMA for self-care provides a more accessible and efficient healthcare management option. It saves my time and eases the process, allowing me to manage many health conditions myself successfully. It saves me from the complications and the effort needed to consult a physician.”*

(I-4)

*“I believe SMA empowers me to manage my health and wellbeing. It’s a critical aspect of maintaining wellbeing during all life stages. Therefore, overall, I consider it an effective approach towards self-care.”*

(I-8)

## 3. Discussion

AMR has emerged as a critical global health concern, posing a severe threat to the effective treatment and control of infectious diseases. The overuse and misuse of antibiotics are among the primary drivers of this crisis, leading to the proliferation of resistant pathogens [29]. A significant contributor to this problem is SMA, which exacerbates the AMR situation by promoting the practice of and by increasing the rate of inappropriate antibiotic consumption [30]. SMA is commonly practiced for minor infections, such as colds and the flu, which are viral in nature and do not require antibiotic treatment. However, there is limited qualitative evidence on the underlying causes and motivators of SMA, particularly in Southeast Asia. Most existing studies, including those conducted in Malaysia, have focused on the prevalence of SMA using quantitative methods, which fail to explore the behavioral and contextual factors influencing this practice [12].

The current study unveils various novel insights from the participants’ in-depth interviews about the non-prescription use of antibiotics, as well as the potential facilitating factors of SMA. Specifically, the interviews provided insights into why participants, who were community-dwelling adults, chose to practice SMA, their opinions on the practice of SMA, where they attained antibiotics from, and how they consumed them. These results revealed a diverse range of views and perceptions concerning antibiotic use in general and related to SMA, which participants primarily employed to treat minor illnesses, like colds, coughs, flu, and fever. A systematic review by the WHO, which focused on Asian countries, found that the population most commonly used non-prescription antibiotics to treat (the symptoms of) colds, flu, and fever [31]; in addition, similar findings were reported in other studies [26,32,33,34,35]. However, there is clear evidence available that antibiotics are ineffective against viral infections (e.g., colds and flu) and provide no health benefits for such illnesses [36,37,38]. In fact, taking antibiotics for mild infections, such as sore throats, bronchitis, or rhinosinusitis, is often unnecessary, as the immune system can typically combat these infections without the need for intervention. Moreover, taking antibiotics does not alleviate symptoms or hasten recovery from these infections [39]. In light of this evidence, using antibiotics for minor illnesses not only fails to benefit the individual taking them but also increases the risk of ABR (both on an individual and population level) and potentially exposes the person to adverse drug effects unnecessarily [40]. Thus, health authorities in Malaysia should raise public awareness of this issue to enhance its appropriate use. A significant factor influencing the practice of SMA among the public is a previous successful experience, which will also contribute to future health-related decisions [41]; these concerns have also aligned with the results of the present research. Participants reported that they would purchase antibiotics directly from pharmacies, as they believed that the same antibiotics would be prescribed for the same illness on future occasions as well. These findings are similar to those found in the study conducted in Saudi Arabia by Alhomoud et al. [42]. Some participants from the current study mentioned a lack of trust in doctors for various reasons, which is a phenomenon that has previously been highlighted by systematic/scoping reviews on the topic [31,43]. On the other hand, some believed that doctors sometimes prescribe unnecessary medications and supplements, purely for economic reasons. Additionally, some of our participants mentioned having health insurance and believed that Malaysian doctors sometimes order unnecessary lab tests to acquire funding.

According to our findings, participants believed that doctors receive incentives from pharmaceutical companies to prescribe pharmaceuticals, including antibiotics [35]. Most people believe obtaining good-quality antibiotics from pharmacies is a better option, as there is a significant issue regarding poor-quality antibiotics (substandard or counterfeit) offered for purchase in Southeast Asia [44,45]. Another key factor that has facilitated SMA among the study population is their cost, and most of the participants agreed that they practiced SMA because antibiotics are expensive, and the high consultation fee for a medical check-up (or control visit) would lead to severe out-of-pocket health expenditures. Furthermore, saving time and avoiding long waiting periods, traffic, and disruption to personal businesses were also cited as major drivers for SMA. The study of Shehadeh et al. from Jordan showed that 60.7% of respondents had poor knowledge about ABR [46]. Furthermore, another noteworthy finding from our study is that most participants could not recall how many times they had self-medicated with antibiotics in the past; however, they indicated that they started using antibiotics when they fell ill and stopped taking them, whether prescribed or not, when they felt better. This aligns with the report of Horvat et al., where nearly half of the study population believed that the consumption of antibiotics may be discontinued once symptoms improved [47]. The debate over whether to stop antibiotic treatment when symptoms improve is longstanding and controversial. While Llewelyn et al. (2017) question the necessity of completing antibiotic courses [48], the WHO and current clinical guidelines still emphasize that full adherence is crucial. Prematurely stopping antibiotics may contribute to the development of ABR [49]. The reason for stopping antibiotics prematurely might be the common misconception that antibiotics are equivalent to painkillers or anti-inflammatory drugs (where the development of ABR is a non-issue). Although studies by Tamma et al. (2023) and Castañeda-García et al. (2020) suggest that shorter courses may be adequate for some self-limiting infections, completing the prescribed regimen remains recommended in most cases [50,51]. This ensures optimal therapeutic outcomes and helps to prevent the development of resistance. Our findings highlight the need for better patient education on antibiotic use. Patients must understand the importance of following prescribed treatments, even if symptoms improve, to avoid complications and to mitigate ABR.

Our qualitative results indicate that participants frequently obtained their antibiotics from pharmacies. However, Malaysian participants mentioned that obtaining antibiotics without a prescription may oftentimes be difficult; to circumvent this, individuals usually have some kind of personal connection with someone employed in a pharmacy. Participants who were not born in Malaysia brought antibiotics from their home countries. They stated that obtaining antibiotics without a prescription is difficult in Malaysia, so they circumvent this by bringing antibiotics from countries they are native to. These results are similar to the study conducted in the United Arab Emirates (UAE) by Al-Kubaisi et al. [52]. Despite the challenges in obtaining non-prescription antibiotics, some Malaysian participants admitted to using leftover antibiotics when needed, primarily due to the difficulty in acquiring them without a prescription. This problem is pervasive globally, as evidenced by studies conducted in Jordan and China [46,47,49,53]. To address these concerns, the public should be encouraged to consult healthcare professionals—by facilitating the availability and access to publicly funded healthcare services to a larger share of the populace—rather than relying on SMA or advice from their social networks on treating their symptoms. Given that pharmacies are the primary source of antibiotics, community pharmacists may play an essential role in advising customers to seek proper medical guidance when trying to acquire antibiotics [54]. Furthermore, pharmacists could inform the public about which ailments do not necessitate antibiotics and convey the risks of consuming antibiotics without a thorough medical assessment. Consequently, healthcare authorities should enforce regulations to improve pharmacy services, ensuring antibiotic quality, distribution, and dispensing. Integrating these qualitative findings may provide a comprehensive understanding of why Malaysian people self-medicate with non-prescription antibiotics. The authors also highlighted an urgent need for formal education programs to inform the lay public about the uses of antibiotics (and the associated risk on both an individual and societal level), which would foster safer usage and a better understanding of antibiotics in the community [55]. Educational campaigns are critical in addressing SMA and AMR. The WHO’s World Antibiotic Awareness Week (WAAW) is an excellent global example that promotes prudent antibiotic use [56]. Malaysia has participated in these efforts, but there is a need to strengthen national campaigns to curb SMA more effectively [24,57]. Countries, such as Sweden and the UK, have implemented successful antibiotic stewardship programs [58], which could serve as models for Malaysia. For instance, Sweden’s “*Strama*” program has successfully reduced inappropriate antibiotic use through targeted public health education, strict prescription guidelines, and monitoring of antibiotic consumption across a plethora of healthcare settings [58]. Similarly, the UK’s “*Keep Antibiotics Working*” campaign has promoted public awareness about AMR and encouraged the public to consult healthcare professionals before using antibiotics [59]. Malaysia could adopt similar strategies by collaborating with community pharmacists and allied healthcare professionals to implement educational initiatives that highlight the dangers of SMA, emphasize the need for completing prescribed antibiotic courses, and encourage patients to consult healthcare professionals before using antibiotics. Additionally, improving access to affordable healthcare services (both diagnostic and therapeutic) could reduce the economic and time-saving factors that drive individuals toward SMA.

There are several limitations to the present study, which should be acknowledged when evaluating its results: Firstly, it relies on self-reported behavior, which may lead to social desirability bias or recall bias. Consequently, participants may underreport or overestimate their antibiotic use, affecting the results’ accuracy. Secondly, the findings’ generalizability may be limited due to Malaysia’s specific cultural, socioeconomic, and health system contexts where the study was conducted. As a result, the insights might not apply to populations in countries with different healthcare settings/healthcare service availability and antibiotic regulations. Additionally, the study does not examine the perspectives of healthcare professionals, such as pharmacists and physicians, on non-prescription antibiotic use. Including their insights could offer a more comprehensive understanding of the issue and contribute to shaping more effective public health strategies. Finally, the study does not consider the quality and potency of antibiotics obtained without a prescription, which could also influence their effectiveness, the development of ABR, and the associated health risks regarding their unnecessary use.

## 4. Materials and Methods

### 4.1. Study Design and Setting

The present study employed a mixed-method, sequential explanatory design, combining surveys and in-depth interviews to collect comprehensive data [60]. The study was conducted from December 2019 to March 2020. For the qualitative phase, an interpretivism paradigm was applied, particularly when exploring complex topics, like SMA. This approach focuses on understanding social phenomena through individual interpretation and meaning-making; this approach aligns with the constructivist/interpretivism framework [61]. The results from the quantitative phase have already been published previously [27]. Following the completion of the first phase of the study, the second qualitative phase was also conducted in Kuala Lumpur, Malaysia, to expand and interpret the findings further. Kuala Lumpur is the capital city of Malaysia and has an estimated population of 1.78 million, with an area of 243 km^2^ [62]. Kuala Lumpur has a population density of 6696 inhabitants per square kilometer (17,340/sq mi), and it is the most densely populated administrative district in Malaysia [62].

### 4.2. Study Population, Selection of Participants, and Inclusion Criteria

**Figure 1:**
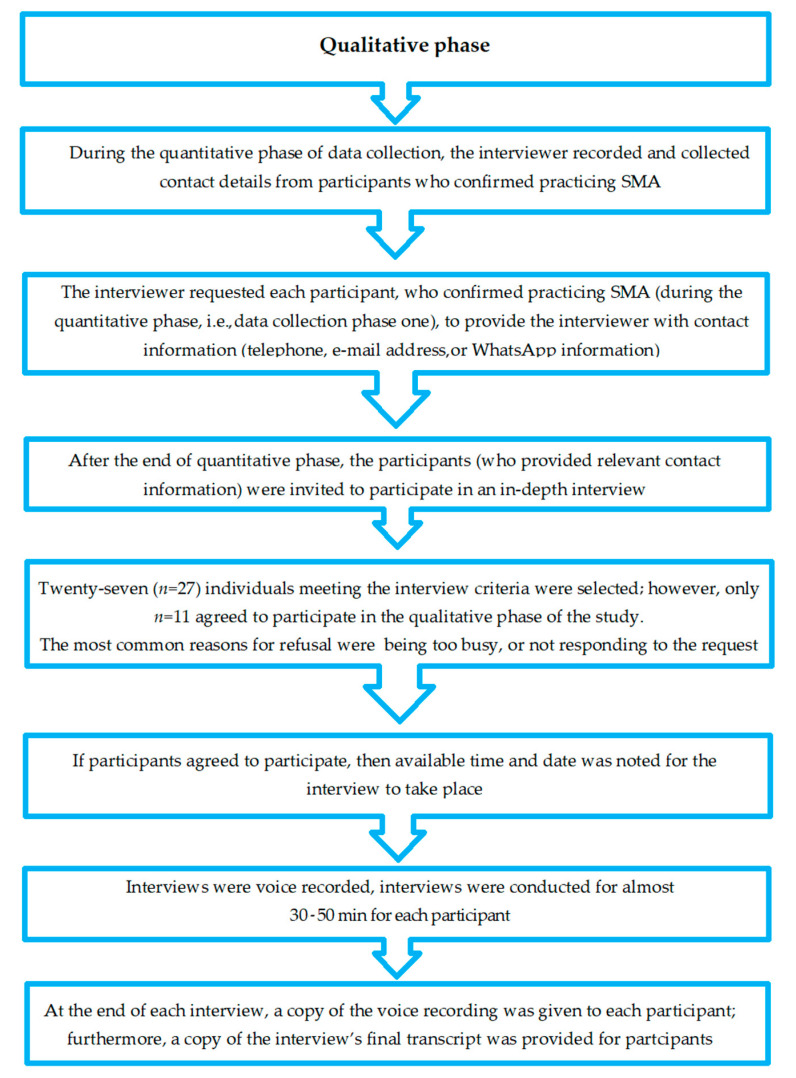
Data collection process for the qualitative, semistructured interviews on SMA.

The current study was conducted in two phases. Participants for the second phase (qualitative) were chosen from those who took part in the first phase (quantitative) [27]. In the quantitative phase, interviewers asked participants who admitted to practicing SMA (*n* = 72) to share their contact information, such as phone numbers, email addresses, or WhatsApp details. This allowed researchers to follow up with them for the second phase of the study. Of the *n* = 72 participants, *n* = 27 provided their contact information and agreed to participate in semi-structured interviews for the qualitative phase. Thus, only those participants who consented to the qualitative study were included in the final analysis. This purposive sampling method helped ensure that participants in the qualitative phase had relevant experience with SMA, supporting the study’s objectives. After the first phase ended, *n* = 27 participants were invited to participate in the qualitative phase; however, only *n* = 11 agreed to participate. Semi-structured interviews were then conducted with these 11 participants (Figure 1). Upon agreement, the preferred times and dates for the interviews were recorded. The study carefully documented the number and key characteristics of participants, which were essential to the research. The participant pool included individuals from diverse demographics, such as age, gender, highest level of educational attainment, and occupation, to ensure a well-rounded exploration of the topic. Participant engagement throughout the research process was closely monitored, including their active involvement during interviews. However, participants did not have a direct role in data analysis or reporting, which helped maintain transparency and the overall thoroughness of the study [63]. The sample size was determined by reaching saturation, the point at which no new information was being gathered [64]. The in-depth interviews were conducted in a convenient and quiet public place in Kuala Lumpur, with the duration ranging between 30–50 min, where no other person was present except the researcher and the participant. Each interview was voice recorded, and notes were also taken during the interview when necessary. At the end of each interview, a copy of the voice recording was given to each participant; furthermore, a copy of the interview’s final written transcript was provided for the participants. The hallmarks of the data collection process are shown in Figure 1.

### 4.3. Development of the Interview Guide, Interview Questions, and In-Depth Interviews

The interview questions for the semistructured interviews were developed and confirmed by academic researchers (three females and two male) (S.J. PhD, C.S.Z. PhD, N.S.A.R. PhD, M.G. PhD, and A.A. PhD) with relevant expertise in qualitative research in the discipline of pharmacy practice and AMR. The interview questions were also assessed to ensure their credibility and accuracy in relation to the study’s aims. Qualitative interviews were conducted in English and included a total of 13 questions (see Supplementary File S1 for the interview guide), including seven main questions and six probing questions. The main questions focused on the participants’ views on SMA and related problems, while the probing questions explored specific details about their antibiotic use.

### 4.4. Interviewer Characteristics and Potential Bias

The interviewer, A.A., held expertise in the field of pharmacy practice and antimicrobial resistance (AMR), which may have influenced the research process. To minimize bias, the interviewer maintained neutrality during the interviews, avoiding leading questions and allowing participants to freely express their views. However, certain assumptions may have arisen due to the interviewer’s prior knowledge and interests in the topic, which could have shaped the framing or interpretation of questions. To prevent potential bias, several steps were taken. A semi-structured interview guide was used to ensure consistency, and the questions were pilot tested to refine any leading or suggestive language. Reflexivity was maintained through a journal to acknowledge and manage personal biases, and participant feedback was encouraged to ensure their responses remained authentic. The process was further strengthened by peer debriefing, where colleagues reviewed the interview methods, and the triangulation of data from various sources helped validate the findings. Additionally, responses were anonymized, and another researcher, blinded to the interviewer’s role, reviewed parts of the data to ensure objectivity. These measures helped safeguard the integrity of the study and minimized the potential influence of interviewer bias on the data collection and analysis processes.

### 4.5. Reliability and Validity

To ensure the reliability and validity of the interview questions, several measures were implemented. Reliability was enhanced through the semi-structured format, which ensured consistency in key topics, while standardized demographic questions provided a solid foundation for categorizing participants. To further improve reliability, inter-coder reliability checks were performed during data analysis. Validity was established through content and face validity, with five subject matter experts (S.J., C.S.Z., N.S.A.R., M.G., and A.D.) reviewing the interview questions to confirm their relevance to the research objectives. Additionally, member checking was employed to confirm the accuracy of participants’ responses, thereby enhancing the credibility of the study. Overall, these strategies ensured that the interview questions effectively measured the intended concepts surrounding SMA.

### 4.6. Interview Questions

The interview, conducted by A.D., was preceded by obtaining a written, signed informed consent form from all participants who agreed to take part in the study. Additionally, an introductory letter was provided to all respondents, which included a brief description of the study objectives and their significance. The interview began by asking participants about their views on SMA, followed by a question about whether they had ever experienced problems related to SMA. Participants were then asked to recall the number of times they had practiced SMA, which antibiotics they had used, and whether they were prescribed or self-purchased. A probing question was added to this, asking participants to remember the names of antibiotics they had used in the past. In the fourth question, participants were asked how they obtained antibiotics without a prescription, and four probing questions were added to explore this further. An interview guide included questions about participants, such as whether they thought it was cost-effective to obtain antibiotics without a prescription, in addition to how quickly participants’ symptoms resolved after taking antibiotics. Furthermore, participants were asked for their overall views on SMA as a good measure for self-care purposes. Overall, the interviews were designed to gather information about participants’ experiences and opinions on SMA, while also exploring specific details about their antibiotic use.

### 4.7. Data Analysis and Thematic Analysis

This study was conducted in accordance with the consolidated criteria for reporting qualitative (COREQ) research statement [65] (Supplementary File S2). Data were analyzed using thematic analysis and a six-phase guide that was used in the current study as a foundation for conducting thematic analysis by following guidelines developed by Braun and Clarke (2006) [66,67]. A total of four researchers (S.J., C.S.Z., N.S.A.R., and A.A.) were involved in excerpting, coding, and the thematic analysis of the qualitative data.

The analysis was conducted manually, following a structured six-phase process. Initially, the transcriptions were thoroughly reviewed to identify significant segments and extract insights. This was followed by generating initial codes systematically across the dataset, allowing for data organization into manageable portions based on known influences on self-medication. The third phase involved categorizing these codes to identify themes and relevant data collection. The refinement of themes occurred in the fourth phase, employing a dual-level analysis for validation facilitated by the senior researchers. In the fifth phase, the focus was on clearly defining each theme and its relationship to the research questions. Finally, the sixth phase centered on composing a narrative that synthesized the findings and provided a comprehensive exploration of the data across themes. Ensuring rigorous standards of reliability and validity for data collection is crucial. Furthermore, the credibility and accuracy of representation are paramount in qualitative studies [68]. To ensure these, all interview transcripts were shared among the researchers involved in coding and theme development. To establish acceptable levels of inter-coder reliability among the team members, each coder independently agreed or disagreed with a coded set of transcripts and then compared the results for internal consistency by applying Cohen’s kappa (κ) [69]; overall, κ was 0.729, which denotes substantial (0.6–0.8) reliability between team members [70].

### 4.8. Ethical Considerations

The study was conducted in accordance with the Declaration of Helsinki (and its later amendments), as well as national and institutional ethical standards. Ethical approval for the study protocol was obtained from the ethical committee at the International Islamic University Malaysia (IREC 2019-004; date of approval: 15 January 2019). Written informed consent was obtained from the respondents before conducting the interview. They were briefed about the research objectives, privacy, and confidentiality of their data. The respondents were made aware that their participation in the research was voluntary and that they could withdraw from the study at any time.

## 5. Conclusions: Implications for Public Health

The current study highlights several implications for health policy and potential future public health strategies related to non-prescription antibiotic use in Malaysia and other Southeast Asian countries. It emphasizes the importance of increasing public education programs that focus specifically on appropriate antibiotic use and the dangers of AMR on both an individual and on a societal level. Such programs should dispel misconceptions about the effectiveness of antibiotics against viral infections and the treatment of symptoms associated with acute respiratory infections and promote the completion of the entire course of prescribed antibiotics. Additionally, health authorities should consider developing policies that strengthen the regulation of antibiotic sales in pharmacies, including stricter enforcement of “prescription-only” policies and the education of pharmacists on their critical role in advising customers about proper antibiotic use during dispensing. Addressing the public’s mistrust in physicians (and the healthcare system as a whole) and the perception of physicians receiving incentives from pharmaceutical companies calls for increased transparency (or limitations/bans) within the healthcare system. Public awareness campaigns that shed light on the role of doctors and the prescribing process may help reduce these negative perceptions. Furthermore, strategies should concentrate on increasing access to affordable and timely healthcare services, as costs and waiting times for medical consultations appear to contribute significantly to non-prescription antibiotic use. Lastly, addressing the issue of leftover antibiotics is essential, possibly through patient education on proper medicine disposal methods and exploring the potential for take-back programs at pharmacies.

The current study concludes that a significant proportion of the Malaysian population may potentially engage in SMA, a widespread practice that seems prevalent, irrespective of socioeconomic status, age, or educational background. This behavior appears to be premised on applying acquired biomedical knowledge in everyday life as a form of self-care. The motivation for SMA largely stems from successful past experiences, influencing future decisions to use non-prescription antibiotics. Distrust in physicians was identified as a notable factor, with participants citing perceived over-prescription of medication by doctors as a reason for their behavior. Economic considerations and time-saving impulses significantly supplement the rationale for SMA. Participants identified cost-effectiveness and circumstantiality, as well as management of time-consuming factors, such as lengthy commutes or owning a business, as primary reasons. To counteract the overuse of antibiotics and prevalent SMA practices, the implementation of robust and efficient public health promotion and health education strategies is crucial. Moreover, effective law enforcement in healthcare-related contexts and regulatory expertise in overseeing compliance with established guidelines for quality assurance and dispensing practices are necessary. The current study’s findings contribute to the understanding of unregulated antibiotic use in Malaysia, offering exploratory evidence in terms of community practices surrounding inappropriate antibiotic use. However, further research is warranted to comprehensively address the issue of unregulated antibiotic use and its implications in Malaysia.

## Figures and Tables

**Table 1:** Socio-demographic characteristics of the study’s participants.

Participant ID	Gender	Age (Years)	Highest Level of Education	Income Category (MYR)	Approximate USD Equivalent	Marital Status	Parent	Type of Occupation
I-1	M	39	HSD	>5000	>1087 USD (>5000 RM)	MA	Yes	Self-employed
I-2	F	32	Undergraduate	<3000	<652 USD (<3000 RM)	MA	No	Self-employed
I-3	M	34	HSD	3000–5000	652–1087 USD (3000–5000 RM)	MA	No	Manual labor
I-4	M	37	Certificate/Diploma	<3000	<652 USD (<3000 RM)	MA	Yes	Self-employed
I-5	M	30	Undergraduate	>5000	>1087 USD (>5000 RM)	MA	Yes	Administrative
I-6	F	37	HSD	>5000	>1087 USD (>5000 RM)	MA	Yes	Skilled labor
I-7	M	36	Certificate/Diploma	<3000	<652 USD (<3000 RM)	MA	Yes	Self-employed
I-8	F	35	Certificate/Diploma	3000–5000	USD652–USD1087 (3000–5000 MYR)	MA	Yes	Manual labor
I-9	F	24	Undergraduate	3000–5000	USD652–USD1087 (3000–5000 MYR)	NMA	No	Self-employed
I-10	M	44	Undergraduate	3000–5000	USD652–USD1087 (3000–5000 MYR)	MA	Yes	Administrative
I-11	M	40	Postgraduate	3000–5000	USD652–USD1087 (3000–5000 MYR)	MA	Yes	Professional

M: male; F: female; HSD: high school diploma; MA: married; NMA: not married; RM: Malaysian Ringgit; USD: US dollar.

**Table 2:** The main themes, subthemes, and codes identified during the study.

Themes	Subthemes	Codes
**Practices towards SMA**	*The purpose of practicing SMA*	Appropriate to treat minor illness Not suitable for a serious illness Specific indications for SMA The convenience associated with SMA SMA speeds up the recovery process from minor ailments SMA prevents serious illnesses from developing
	*Reasons/barriers related to SMA*	Mistrust of physicians Difficulties faced with travelling to receive healthcare Long waiting times at the hospital
	*Most common sources for getting antibiotics to practice SMA*	Community pharmacies and “chemists’ stores” are the main sources of antibiotics for SMA Easy access to purchase antibiotics without a prescription Easy access to purchase antibiotics without old/expired prescriptions
	*Advantages of SMA* (cf. *sourcing antibiotics the “official” route)*	Opportunity to save time and money
**Attitudes towards SMA**	*Attitudes regarding the use of antibiotics*	Frequency in the past Compliance
**Knowledge related to SMA**	*ABR*	Concepts related to ABR, AMR, and drug resistance in general
	*General knowledge regarding the use of antibiotics*	SMA increases my knowledge and confidence related to infectious diseases and their therapy

SMA: self-medication with antibiotics; ABR: antibiotic resistance; AMR: antimicrobial resistance.

## Data Availability

All data generated during the study are presented in this paper and its Supplementary Materials.

## Supplementary Materials

The following supporting information can be downloaded at: https://www.mdpi.com/article/10.3390/antibiotics13111070/s1, Supplementary File S1: English version of the interview guide; Supplementary File S2: Consolidated criteria for reporting qualitative research (COREQ) checklist.
