# Supplementary material for: Understanding the Determinants Influencing Self-Medication with Antibiotics Among Malaysian Residents: A Qualitative Study to Inform Preventive Public Health Strategies
Source: Antibiotics (Basel). 2024 Nov 11;13(11):1070. doi: 10.3390/antibiotics13111070 (PMC11591425; doi:10.3390/antibiotics13111070)
Supplement: Supplementary file 1 [file antibiotics-13-01070-s001.zip › Supplemental file S1.pdf]

Supplementary Material S1: English version of the interview guide used during the study *"Understanding the determinants influencing self-medication with antibiotics among Malaysian residents: a qualitative study to inform preventive public health strategies"*

Introduction:

"Hello, my name is XYZ, and we are conducting a qualitative research study on the subject of self-medication with antibiotics. Thank you for participating in this interview. Before we begin, have you had a chance to review the information sheet, and do you have any questions about the study process?"

Explanation of interview process:

"In this interview, we'll discuss your experiences and perspectives on self-medicating with antibiotics. The discussion will be recorded for accuracy and will last approximately 30-50 minutes. Your participation is voluntary, and you can choose not to answer any question. If there are topics you'd prefer not to discuss, please let me know. All information shared will be kept confidential. Any questions before we start?"

[Start the audio recording]

Demographic information:

- What is your gender?
- What is your age (in years)?
- What is your level of education?
- What is your income (in RM)?
- Are you married?
- Do you have children?
- What is your current occupation?

Views on self-medication with antibiotics:

1. Could you please tell me about your views on self-medication with antibiotics?

Awareness and problems related to self-medication:

2. Have you ever heard of any problems related to self-medication with antibiotics?

Frequency and names of drugs used for self-medication:

3. Do you remember how many times you have self-medicated with antibiotics?

4. Can you recall the names of antibiotics you've used in the past? Were these prescribed by a doctor, or did you purchase them on your own, without a prescription?

Source of antibiotics:

5. Was it from a doctor, or can you buy antibiotics easily on your own?

6. How and where do you usually buy antibiotics without a prescription?

7. Could you share some previous experiences of obtaining antibiotics without a prescription?

8. Is it from a chemists’ shop, pharmacy, or elsewhere?

Dosage adherence and use of leftover stock:

9. Have you ever used a leftover antibiotic stock?

10. Were there any cases in the past when you did not complete the dosage regimen?

Financial aspects of self-medication:

11. Is it financially beneficial for you in any way to obtain antibiotics without a prescription from a pharmacy or a friend?

Symptom resolution and adverse effects:

12. Could you please tell how quickly your symptoms typically resolve after taking antibiotics?

13. Have you ever experienced any adverse drug effects or other effects when using antibiotics on your own accord?

Views on self-medication for self-care purposes:

14. Could you please share your views on self-medication as a step for self-care?

Closing:

1. Is there anything important that we haven't discussed, and you feel should be included in our study?

2. Do you have any recommendations for others who might be interested in participating in this study?

"Thank you for sharing your experiences and insights. Your input is invaluable to our research."

[Stop the audio recording]
